# An underexplored pathway to life satisfaction: The development and validation of the synchronicity awareness and meaning-detecting scale

**DOI:** 10.3389/fpsyg.2022.1053296

**Published:** 2023-01-16

**Authors:** Pninit Russo-Netzer, Tamar Icekson

**Affiliations:** ^1^Department of Advanced Studies, Achva Academic College, Aurugot, Israel; ^2^Department of Counseling and Human Development, University of Haifa, Haifa, Israel

**Keywords:** synchronicity experiences, synchronicity awareness and meaning-detecting scale, search for meaning, presence of meaning, individual differences, life satisfaction, well-being

## Abstract

**Introduction:**

Synchronicity refers to the psychological process of meaningful coincidences. The present study aimed to build and expand upon a model of synchronicity awareness and meaning-detecting (REM)—receptiveness (R) as a precondition for an exceptional encounter (E) triggering emotions and meaning-detecting (M)—by assessing the prevalence of the phenomenon and its associations with well-being.

**Methods and Results:**

Results from two studies reported here employing adult community samples (*N* = 198 and *N* = 440) demonstrate coherent, replicable structure and good internal reliability for a 35-item, two-factor Synchronicity Awareness and Meaning-Detecting (SAMD) Scale. Synchronicity awareness (SA) and meaning-detecting (MD) scores were significantly associated with some of the Big-5 personality dimensions and tolerance for ambiguity, as well as with search for and presence of meaning. Furthermore, process mediation models showed: (a) synchronicity awareness mediated the relationship between search for meaning and meaning-detecting, and (b) optimism and presence of meaning in life partly mediated the relationship between meaning-detecting and life satisfaction.

**Discussion:**

The findings suggest the importance of synchronicity experiences and hold important conceptual and practical implications for understanding processes of meaning making from unexpected events and their potential contribution to individuals’ well-being.

## Introduction

Synchronicity has been defined by Jung (1969) as unpredictable occurrences of meaningful coincidence. Synchronicity refers to unusual and meaningful coincidences linking the internal and external worlds of the individual. In essence, synchronicity experiences reflect “the coincidence of events in space and time as meaning something more than mere chance” (Jung, 1950/1997, p. 25). In the decades since Jung introduced the concept of synchronicity, interest in the topic has significantly grown (Main, 2011; Hocoy, 2012; Sacco, 2019). Clinical case studies have demonstrated that an acknowledgment of synchronicity is beneficial in therapeutic settings (Connolly, 2015; Roxburgh et al., 2015), as well as in understanding career pathways and processes (e.g., Guindon and Hanna, 2002). Yet, systematic empirical findings regarding the prevalence of this phenomenon and its association with meaning in life and well-being among nonclinical populations remain underexplored. Moreover, assessment tools suggested to explore this phenomenon have failed to reflect the full range of the experience. In line with this, the purpose of the present study was twofold: (1) to extend previous attempts by developing a new scale to assess individual differences in the capability to be aware and make sense of synchronicity experiences; and (2) to explore potential links between the awareness and meaning-detecting of synchronicity experiences, meaning, and well-being.

Following the Jungian view, synchronicity reflects a holistic experience, in which an external experience has meaning when it is connected to a person’s inner world (Jung, 1950/1997). Such interconnectedness between the inner and external experience relies on subjective interpretation of events. Indeed, research thus far indicates a great variance in the reported occurrence of such events. An estimated 22 to 84% of the population reported experiencing synchronicity at least once (Henry, 1993; Fach et al., 2013; Roxburgh et al., 2015; Sacco, 2019)].

Preliminary quantitative reports pointed out individual differences in the experience of synchronicity and coincidence (Bressan, 2002; Coleman et al., 2009; Fach et al., 2013). Along these lines, Bressan (2002) suggested that awareness of meaningful coincidence can be considered a trait-like characteristic. Unger et al. (2021) also found that “people show marked and temporally stable individual differences concerning the frequency of meaningful coincidences perception in everyday life” (p. 1). More specifically, the tendency to detect such coincidence was found to be related to some of the Big Five personality traits, referential thinking, religious commitment, and faith in intuition (Coleman et al., 2009), or belief in the paranormal (Bressan, 2002).

In sum, a few studies suggest that individuals appear to differ in their tendency to notice coincident events (e.g., Roesler, 2018) as well as in their inclination to make sense of them (e.g., Coleman et al., 2009). However, to our knowledge, none of the few studies to date on the topic have clearly differentiated between the awareness of such events and the process of making sense of them. Moreover, little is known about the relationship between these constructs and other personality characteristics or with life satisfaction.

### Assessment of synchronicity experiences

Increased attention to the phenomenon of synchronicity experiences led to various attempts to estimate their prevalence (e.g., Henry, 1993; Fach et al., 2013). Despite their contribution to the development of better understanding of the frequency of such experiences, these attempts were limited in four ways. First, previous studies mostly focused on specific populations, such as reports on chance events in careers (Guindon and Hanna, 2002; Bright et al., 2005; Krumboltz et al., 2013), in the context of psychotherapy and clinical settings (Marlo and Kline, 1998; Connolly, 2015; Roesler, 2018), or self-selected participants interested in “weird coincidences” (Coleman et al., 2009). Second, previous studies mostly used qualitative methodologies and small samples to explore the nature of the phenomenon (e.g., Hanson and Klimo, 1998; Connolly, 2015; Roxburgh et al., 2015; Russo-Netzer and Icekson, 2020). Third, some explorations relied on reports that only addressed the phenomenon as a part of other experiences. For example, Fach et al. (2013) explored meaningful coincidences as part of their study of exceptional experiences, along with other types such as out-of-body experiences. Fourth, the few studies that explored individual differences in the tendency to report synchronicity experiences paid less attention to the possible links between this phenomenon and well-being (e.g., Bressan, 2002; Coleman et al., 2009; Fach et al., 2013; Roesler, 2018). Together, these limitations suggest that there is a need for more nuanced and sensitive measures of synchronicity experiences in the general population. Moreover, exploring the potential mechanisms underlying this phenomenon – awareness of the experience and the meaning-detection of it – and the relationships of these components to well-being may enable a more holistic understanding of the construct.

Following Jung (1969) conceptualizations of synchronicity experiences and a phenomenological analysis of in-depth interviews, a model was recently offered to characterize the experience of synchronicity and meaningful coincidences (Russo-Netzer and Icekson, 2020). The model (REM) refers to three major building blocks: receptiveness (R), or increased attention and openness to both a person’s internal and external world, which is viewed as a precondition for an exceptional encounter (E), a sudden unexpected event that corresponds with a person’s inner state of mind, triggering memorable and distinctive emotions, and meaning-detecting (M), a conscious process of connecting the event to a person’s life narrative. The first aim of the present study was to build on this model and to validate a tool to assess individual differences in the experience of synchronicity, while measuring both aspects of the experience (i.e., awareness and meaning-detecting).

Synchronicity experiences have been reported as emerging more frequently around periods of emotional intensity or major life transitions, such as births, deaths, and marriage (Coleman et al., 2009). Moreover, synchronicity experiences have been viewed as a possible vehicle for personal transformation (Jung, 1969; Main, 2011), as well as for individuals’ growth (e.g., Russo-Netzer and Icekson, 2020). However, the understanding of the mechanisms underlying the relationship between these experiences and well-being is still limited. This is due to the rather narrow and anecdotal nature of case studies in therapeutic and career development settings that informed previous attempts (Guindon and Hanna, 2002; Connolly, 2015; Roxburgh et al., 2015). Therefore, this study also aims to explore potential links between these aspects of synchronicity experiences, meaning, and well-being.

### Synchronicity, meaning, and life satisfaction

Life satisfaction (LS) is often conceived as a general, overarching well-being indicator (e.g., Lounsbury et al., 2005), evaluating individuals’ sense of well-being, measuring their overall satisfaction with life (Diener, 1984). Psychological study of the human experience of LS has received much attention in recent decades (Emerson et al., 2017), suggesting various beneficial outcomes (e.g., Diener et al., 2017; Lombardo et al., 2018). Yet, more research is needed to uncover the psychological mechanisms that contribute to higher LS (Diener et al., 2017).

One potential mechanism, according to existential and humanistic theories, is the search by individuals for meaning in their lives. The process of searching for meaning in life is considered by some theorists as healthy and positive (Frankl, 1963). For example, search for meaning was found to be positively related to LS in some studies (e.g., Steger et al., 2008; Datu, 2015; Russo-Netzer, 2019; Abu-Raiya et al., 2021), as well as with positive outcomes such as open-mindedness, ambition, and absorption (Steger et al., 2006, 2008).

**Figure 1:**
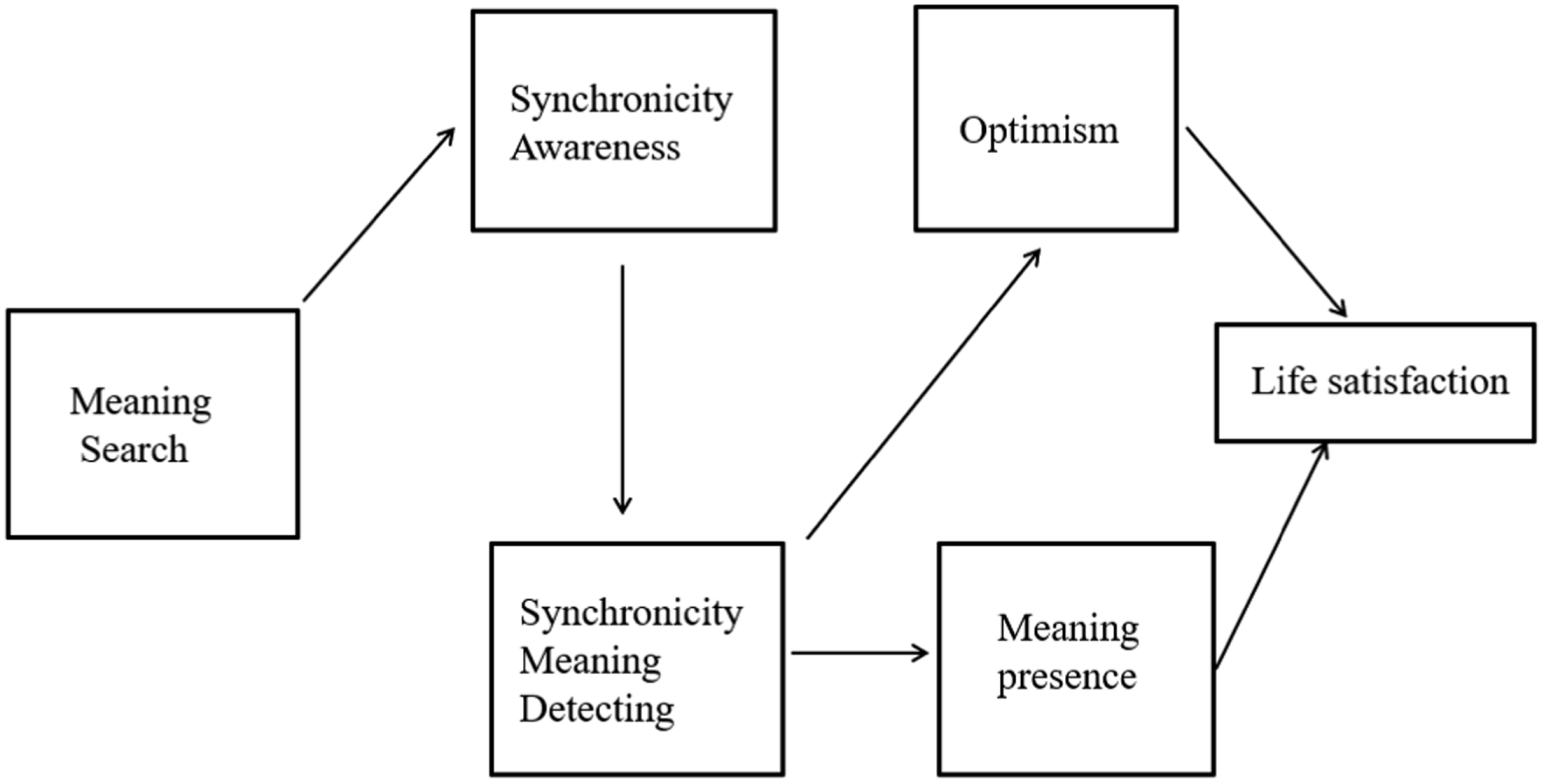
The proposed theoretical model.

Yet, other studies have found that searching for meaning is associated with less LS (e.g., Park, 2010) and greater anxiety, depression, and rumination (e.g., Steger et al., 2008; Yek et al., 2017). Thus, the relationship between search for meaning and well-being indicators appears to present a complex and multifaceted picture, suggesting that the interplay between these constructs may require further unpacking (*cf*. Russo-Netzer, 2019). The present study aimed at extending knowledge on the relationship between search for meaning and LS, pointing to the role of synchronicity awareness and meaning-detecting as possible mediators (see Figure 1).

Studies suggest that the search for meaning may function as a state of mind or a schema that allows individuals to identify information related to meaning in life (Steger et al., 2011). Since James (1890/1950) assertion that “my experience is what I agree to attend to” (p. 402), it has long been acknowledged that our attentional choices may shape our experience (e.g., Driver, 2001). For example, the human need to experience life as coherent motivates individuals to prefer clarity and structure over ambiguity and uncertainty (e.g., Heine et al., 2006). Along this line, higher rates of meaning in life were reported following an exposure to coherent stimuli compared to random ones (Heintzelman et al., 2013). Moreover, when confronted with a threat to their sense of personal control, individuals tend to detect patterns in arbitrary displays (e.g., Whitson and Galinsky, 2008). It thus can be suggested that when the search for meaning is expressed by actively being aware of synchronistic experiences and making sense of them, the search for meaning may be expected to be associated with benevolent outcomes. In a recent qualitative study, synchronicity experiences were reported as emerging from an active “searching” mindset that the participants adopted: “Although coincidences are unpredictable, participants described the state of attentiveness to such moments as paradoxically inviting such experiences, as turning them from invisible to apparent in life” (Russo-Netzer and Icekson, 2020, p. 5).

Experiences of unpredictable happenings may temporarily shake and challenge individuals’ sense of certainty, predictability, and control in life (Heine et al., 2006; Proulx and Heine, 2008). However, building on the meaning-as-information approach (Heintzelman and King, 2014) and the idea that identifying consistent connections in the environment is a key for survival (Geary, 2004), when individuals are capable of making sense of such happenings, it may open up opportunities for greater sense of meaning in life and a positive perspective on the future.

#### Presence of meaning mediates between synchronicity meaning-detection and life satisfaction

It has long been acknowledged that experiencing meaning in life contributes to the perception of satisfaction with one’s life as an activating mechanism to optimal living. Extensive research has provided evidence that the presence of meaning is beneficial to a host of well-being indicators (e.g., Park, 2010; Steger, 2012; Czekierda et al., 2017). Moreover, recent studies suggest that meaning-cultivating activities (i.e., prioritizing meaning on a daily basis) are associated with a greater sense of meaning in life, which in turn is positively related to various well-being indicators, such as LS (Russo-Netzer, 2019; Russo-Netzer and Shoshani, 2020). Thus, when individuals experience unpredictable happenings as coherent and meaningful, it may be associated with greater sense of meaning in life, which in turn may contribute to increased LS.

#### Optimism mediates between synchronicity meaning-detection and life satisfaction

Optimism can be defined as a tendency to hold generalized positive expectancies even in the face of adversity (Carver and Scheier, 1994). According to this view, individuals with higher optimism expect good things to happen in the future and therefore actively strive to achieve their goals. Following expectancy-incentive models, desired goals direct and motivate our behavior. If an individual has an internal, purposeful goal, which is believed to be obtainable, the result is engagement of effort and higher satisfaction with life (Carver and Scheier, 2014).

Indeed, more than three decades of research have demonstrated the existence of strong positive ties between optimism and LS, suggesting that the inclination to expect good things in the future is highly associated with individuals reporting a satisfying life (e.g., Carver and Scheier, 1994; Busseri, 2013; Zhang et al., 2014). For example, in a meta-analysis conducted across 50 studies with 19,831 participants, Alarcon et al., 2013 found an aggregate effect of 0.43 between optimism and LS. Over and above the ample number of correlational studies, longitudinal studies also suggest that optimism predicts LS over time (e.g., Daukantaitė and Zukauskiene, 2012; Layous et al., 2013).

Moving one step further, according to the self-concordance model, self-concordant goals are ones that represent people’s authentic interests and values (e.g., Sheldon and Elliot, 1999). Greater self-concordance is experienced when individuals engage in meaning-making activities, such as setting meaningful goals. Hence, greater engagement in meaningful activities leads to greater positive expectancies regarding the future (i.e., optimism), which in turn enhances well-being. Indeed, several previous studies documented mediational effects of optimism in the relationships between meaning-oriented constructs and activities and well-being (Ho et al., 2010; Krok and Telka, 2019). In the same vein, when individuals experience unpredictable happenings as coherent and meaningful, it may be associated with positive expectations toward the future (i.e., optimism), which in turn may contribute to increased LS.

Taken together, our model suggests that individuals who search for meaning are more prone to be aware of synchronicity experiences and notice them and that detecting meaning in synchronicity experiences enhances both optimism and presence of meaning, which eventually are positively associated with higher LS (see Figure 1).

### Overview of the present studies

We assume that people differ in their tendency to detect synchronicity experiences and make sense of them. However, no valid assessment tool has yet to be offered and validated for this purpose. The development of such an assessment tool is necessary to better understand the prevalence of the phenomenon in various populations, as well as to explore its potential relationships with other psychological constructs. Thus, the present studies make three major contributions.

First, they extend previous attempts to explore the scope and prevalence of the phenomenon of synchronicity awareness among nonclinical populations (e.g., Bright et al., 2005; Fach et al., 2013). Second, they offer a new and valid measure for the awareness and meaning-detection of synchronicity experiences to explore potential individual differences that may delineate directions for future research and practice. Third, they contribute to better understanding of the paths that may cultivate well-being.

The two studies were conducted to explore the factorial structure and validity of the Synchronicity Awareness and Meaning-Detecting (SAMD) Scale, comprised of two subscales. Study 1 involved three main steps. Step 1 tested and refined a preliminary items pool of the scale. Step 2 assessed its structural validity and internal reliability. Step 3 assessed its discriminate and convergent validity, comparing it with related constructs of individual differences. It was predicted that both synchronicity awareness and meaning-detecting would be positively related to openness to experience and tolerance for ambiguity, as well as with the search for and presence of meaning in life. Study 2 further validated the internal structure of the scale in an independent and larger sample. In addition, it examined the association between this scale and well-being variables. More specifically, search for meaning was expected to be positively associated with synchronicity awareness (*a*), which leads to higher synchronicity meaning-detecting (*b*). Higher tendency to detect meaning in synchronicity experiences enhances both presence of meaning in life (*c*) and a sense of optimism (*d*), and each contributes to enhanced life satisfaction (*e* and *f*). See Figure 1 for more details.

## Materials and methods: Study 1

The purpose of Study 1 was to develop and test a synchronicity measure. Its factor structure and internal consistency were examined, as well as its associations with conceptually related individual differences constructs.

### Participants

Appropriate sample size for the exploratory factor analyses was determined using the relatively strict ratio of 10:1 subject to item (Costello and Osborne, 2005; Carpenter, 2018). The overall sample consisted of two independent subsamples: the first was a convenient sample and the second a representative sample of the Israeli Jewish population. The integrated sample consisted of 410 Israeli adults with a mean age of 39.22 years (*SD* = 15.67). Approximately 60% of the sample (*n = 244*) were women, 35.4% had a BA degree, and 18.8% had an MA degree or higher. As for marital status, 35.4% were single, 55.6% were married, and 9% were divorced or widowed. Most of the participants were secular (53.9%) and the rest reported various levels of religiosity. Supplementary Table S1 presents the demographics of each of the samples.

### Procedure

Participants were recruited *via* two main vehicles. The first was an online panel. The study utilized an Israeli paid survey platform acknowledged by the Israeli Bureau of Statistics as representing the Israeli population. The panel consists of more than 50,000 people over the age of 18 who signed up to participate in paid internet surveys. Recently, online panels have become a common way to target and reach respondents in social science research (Steelman et al., 2014; Toder-Alon et al., 2018). Participants from the panel received compensation of $5 for filling in the questionnaire. The second vehicle was various mailing lists and websites targeting the general public as well as university students. These participants received no compensation for participating in the study.

All participants completed a series of online questionnaires. Prior to filling out the questionnaires, all participants provided a signed informed consent, which specified the purpose of the research, its procedures, and the voluntary nature of participation. Participants were guaranteed anonymity, and no disclosure of personal details was required. The study was approved by the IRB in the first author’s university.

### Measures

#### Synchronicity awareness and meaning-detecting

This scale was developed specifically for the present study in order to explore the extent to which individuals are aware of the occurrence of synchronicity events in their lives and make sense of them. In order to ensure content validity, the preliminary item pool was developed based on an extensive review of existing conceptual models and surveys. Specifically, the REM (receptiveness, emotion-evoking experiences, and meaning-detecting) model, emerging from the Russo-Netzer and Icekson (2020) qualitative-phenomenological study, informed the creation of the scale. First, we employed a “bottom-up” approach using a content analysis of former in-depth interviews of synchronicity experiences (Russo-Netzer and Icekson, 2020), from which corresponding items were developed. After the preliminary generation of items, and as suggested by Carpenter (2018), we asked for experts’ feedback. Two clinical psychologists who were also experienced researchers (PhDs) provided feedback regarding item quality and how well each item reflected the suggested subscale.

The SAMD scale is comprised of two subscales: (a) synchronicity awareness (SA), and (b) synchronicity meaning-detecting (MD). The SA subscale referred to awareness of the occurrence of synchronicity events. It involved 10 items using a 6-point scale (0 = never, 1 = once, 2 = twice or more, 3 = rarely, 4 = often, 5 = all the time) and included the following instructions: “In our daily lives, surprising and unlikely events may occur. Below are examples of such possible occurrences. For each example try to remember whether you experienced it and indicate the degree of frequency in your life” (e.g., “I thought about a person and he\she contacted me unexpectedly shortly afterwards”).

The MD subscale referred to the meaning detected in the synchronicity events or experiences. It involved 22 items using a 7-point scale (1 = not at all to 7 = to a high degree) and included the following instructions: “Read carefully each of the following items and indicate the degree to which each of these items best describes you” (e.g., “I believe that listening to internal and external occurrences enables new discoveries”). Since the first dimension referred to the *frequency* of specific events and the second dimension to the *subjective perception* of such events, a different response format (i.e., 6- and 7-point scales) and different number of items were adopted for each dimension.

The measure was translated into English using back-translation independently by both the authors and two native English speakers who are bilingual in Hebrew and English (see Table 1 for items and psychometric information). The translation was done in accordance with guidelines from the International Test Commission (ITC; Hernández et al., 2020).

**Table 1:** Exploratory structural equations modeling: factor loadings of the SAMD scale items.

SAMD Scale		
Item	Item loading		Synchronicity awareness	Synchronicity meaning-detecting
I felt that I was “in the right place, at the right time”	**0.721**	0.192
I ran into something or someone that I thought about in an unexpected place	**0.718**	0.086
I ran into a situation or a personal encounter that opened up new opportunities	**0.674**	0.159
I experienced an extraordinary synchronization of thought, behavior. Or words with another person	**0.672**	0.258
I received an answer to a certain need I had in an unpredictable way (e.g., a partner, a job offer, or an apartment)	**0.662**	0.216
I thought or dreamt about a person and then I met him\her somehow in the real world shortly afterwards	**0.651**	0.171
I thought about a person and he\she contacted me unexpectedly shortly afterwards	**0.640**	0.241
I thought about a particular idea and then I saw it as an external image (e.g., a quote, an ad, or a song)	**0.604**	0.251
While in nature, I felt a strong sense of connection to the world	**0.536**	0.317
I believe that unexplained events enable new discovery and development	0.212	**0.781**
I find signs of inner feelings in the external stimuli in the world around me	0.244	**0.763**
I find meaning in unexplained occurrences	0.257	**0.739**
I believe that listening to internal and external occurrences enables new discoveries	0.342	**0.734**
I sometimes feel that the environment “sends” me signals	0.308	**0.729**
Following experiences I’ve had, I have a sense of deep knowing of myself and the world	0.259	**0.724**
I am open to experiences that may not necessarily be explained by reason or causality	0.206	**0.722**
I tend to be attentive to intuition in my everyday life	0.242	**0.673**
I am curious about surprising events in my life	0.178	**0.658**
I walk around in the world with a sense of awe and wonder from the opportunities and surprises that the world has to offer	0.258	**0.651**
It happens that things related to issues I am concerned with suddenly appear more in my everyday life	0.275	**0.622**
I believe that there is something to be learned from any event in life	0.050	**0.621**
I tend to be attentive to physical and bodily sensations (e.g., goosebumps, pain, sense of warmth)	0.118	**0.588**

Target factor loadings are in bold.

#### Meaning in life

This scale was used to assess the search for and presence of meaning in the individual’s life, with “search for” and “presence of” representing two subscales of the overall measure (Steger et al., 2006). Both subscales were rated using a 1 (absolutely untrue) to 7 (absolutely true) Likert scale. The present study used the validated Hebrew version of this questionnaire (Littman-Ovadia and Steger, 2010). The search subscale is comprised of five items (MLQ-S; e.g., “I am looking for something that makes my life feel meaningful” and “I am seeking a purpose or mission for my life”), and Cronbach’s α coefficient = 0.89, 95% CI [0.88, 0.91], McDonald’s ω coefficient = 0.89, 95%CI [0.87, 0.91]. The presence of meaning subscale is comprised of the remaining five items of the measure (MLQ-P; e.g., “I understand my life’s meaning” and “My life has no clear purpose”), and Cronbach’s α coefficient = 0.87, 95%CI [0.85, 0.89], McDonald’s ω coefficient = 0.87, 95%CI [0.84, 0.89].

#### Big five personality traits

A short version of the standard BFI-10 (Rammstedt and John, 2007) and BFI-44 (John and Srivastava, 1999) was used to evaluate the Big Five personality traits: Agreeableness/Antagonism, Conscientiousness/Lack of direction, Emotional stability/Neuroticism, Extraversion/Introversion, and Openness/Closedness to experience. The Rammstedt and John BFI-10 scale includes two items for each of the five personality dimensions (e.g., Neuroticism: “gets nervous easily”; Extraversion: “is reserved”; Openness: “has an active imagination”; Agreeableness: “is generally trusting”; and Conscientiousness: “tends to be lazy”). Participants were asked to respond to each item indicating whether they agreed or disagreed with the statement, using a 5-point Likert-type scale, from 1 (strongly disagree) to 5 (strongly agree). The Hebrew translation of the BFI-10 was found to be reliable and valid (e.g., Berkovich and Eyal, 2019).

#### Ambiguity tolerance

This scale was used to assess the individual’s cognitive tolerance range (from aversion to attraction) for situations that are unfamiliar or ambiguous (McLain, 2009). It included 13 items, such as “I avoid situations that are too complicated for me to easily understand” and “I find it hard to make a choice when the outcome is uncertain,” on a 5-point scale, from 1 (strongly disagree) to 5 (strongly agree). The Hebrew translation of the MSTAT-II scale was found to be reliable and valid (e.g., Nadler and Braunstein-Berkovitch, 2017). Cronbach’s α coefficient = 0.74, 95%CI [0.70, 0.78], McDonald’s ω coefficient = 0.69.

### Data analysis

In order to test and refine a preliminary items pool of the two subscales, an exploratory factor analysis (EFA) was conducted. We followed the guidelines for developing and validating scales suggested by Boateng et al. (2018) and Carpenter (2018). First, Varimax rotation with Kaiser normalization was used, given that the two suggested factors refer to rather independent and distinct dimensions of the phenomena at hand. Specifically, the EFA was obtained using three methodologies: (1) Eigenvalue (EV) > 1; (2) Scree plot – random errors tend to converge on a linear line, data points beyond the break point “the knee” are considered actual factors (Auerswald and Moshagen, 2019); and (3) Parallel analysis – using a randomized data set with equal number of variables and equal number of observations to obtain parallel EVs, only actual EVs that are larger than the parallel ones are considered actual factors. The reference eigenvalues were calculated using the mean and 95th percentile of all eigenvalues generated by principal component analysis of the random data set (Hayton et al., 2004).

The final factors were derived using acceptable data reduction techniques, including the following guidelines: minimal loading in one item of 0.40, items are not loaded above 0.40 in more than factor, there are not loading gaps smaller than 0.20 between factors, and items have commonalities >0.50 (Yong et al., 2013; Howard, 2016). In the next step, internal reliabilities of the two subscales were assessed using both Cronbach’s alpha and total omega (McDonald, 1999; McNeish, 2018). Finally, in order to assess the discriminate and convergent validity of the new scale, pairwise Pearson correlations with Big Five personality dimensions, tolerance for ambiguity, and the search for and presence of meaning in life were calculated and compared.

## Results: Study 1

We started the EFA with a total of 32 items, 10 of which referred to the SA subscale and 22 to the MD subscale. The combined item pool was analyzed using maximum likelihood technique (Varimax rotation with Empirical Kaiser normalization (Braeken and Van Assen, 2017) to explore the factorial structure of the newly developed SAMD scale. Parallel analysis suggested two actual factors in the data.

**Figure 2:**
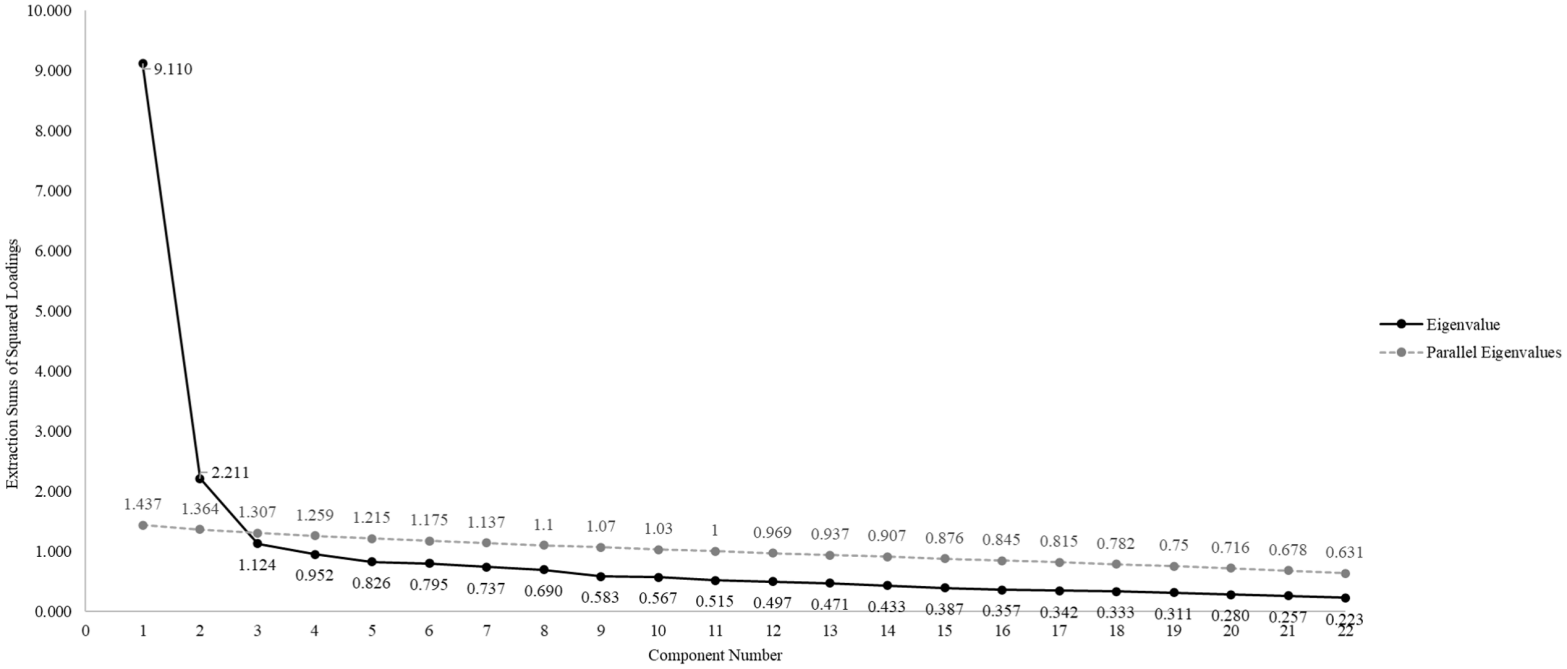
Scree plot with original and parallel analysis Eigenvalues.

Based on the two-factor structure that was found through both iterations, we employed another round of exploratory factor analysis (See Scree-plot with original and parallel Eigenvalues on Figure 2). At this stage, data reduction was performed. Overall, 10 items were dropped over the course of 5 iterations. Eight items were dropped due to double loadings on both factors, while two items were dropped for low loadings on either factor. Based on the aforementioned analyses, the remaining 22 items converged into two factors (see Table 1 for details).Extraction (unrotated) loading for SA was 2.21 and its internal reliability was good as well, Cronbach’s McDonald’s ω coefficient = 0.86. Loading for MD was 9.11 and its internal reliability was good, Cronbach’s Together, both factors accounted for 51.46% of the variance in items and the Pearson correlation between both factors was significant, moderate, and positive: α=0.86, $95\%CI\;\left[0.84,0.88\right]\text{,}$ α=0.93,95%CI0.920.94,McDonald'sωcoefficient=0.93. Together, both factors accounted for 51.46% of the variance in items and the Pearson correlation 329 between both factors was significant, moderate, and positive: r=0.59,p<0.001 .

**Figure 3:**
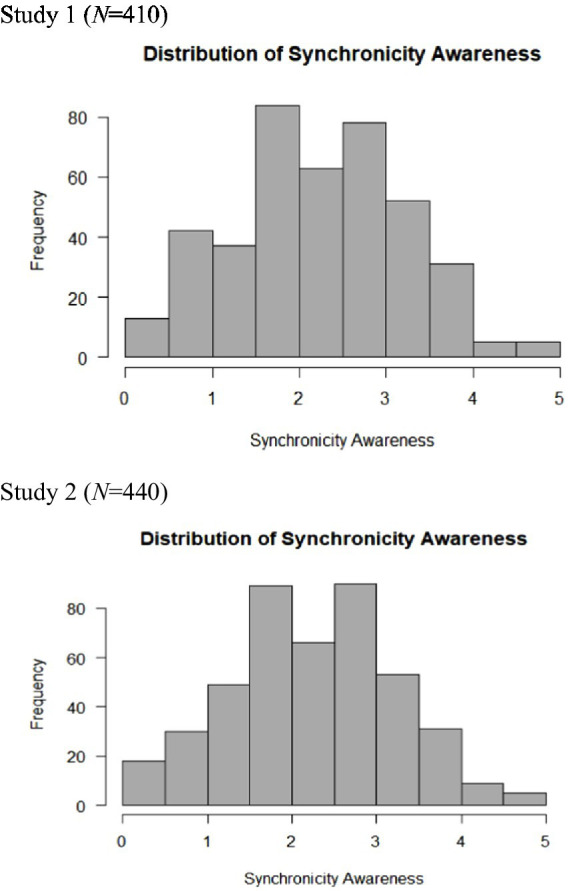
The distribution of synchronicity awareness.

**Figure 4:**
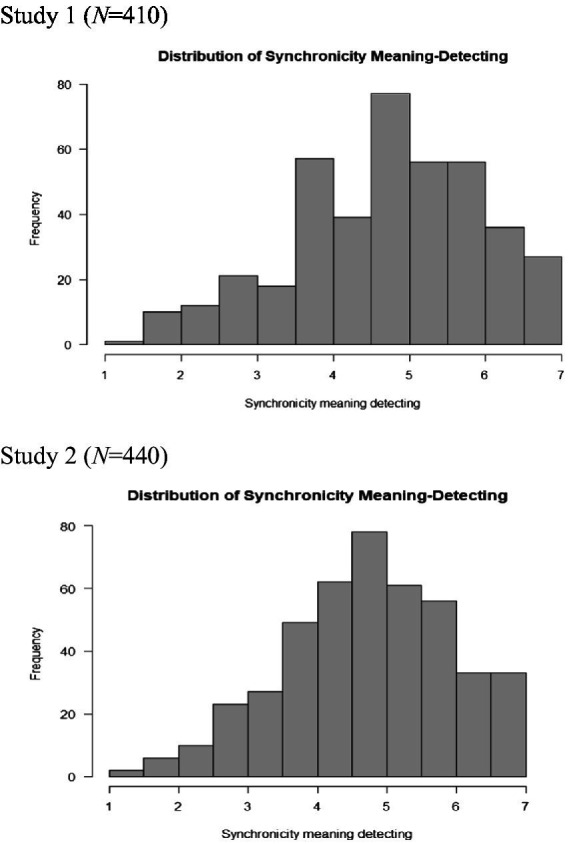
The distribution of synchronicity meaning-detecting.

As for the prevalence of synchronicity experiences, all participants reported they experienced at least one or more such encounters. As Table 2 shows, when asked to estimate the frequency of synchronicity experiences in their daily lives on a scale from “never” (0) to “all the time” (5), the average response of the sample ranged between “twice or more” (2) to “rarely” (3) (*M* = 2.26, *Mdn* = 2.22, *Mode* = 2, *SD* = 0.99, *range* 5, *skewness* = 0.02, *Kurtosis =* −0.39). The distribution of synchronicity awareness is presented in Figure 3. The results suggest that synchronicity experiences are quite common, yet the tendency to be aware of such experiences varies between individuals. The distribution of synchronicity meaning-detecting was *M* = 4.73, *Mdn* = 4.85, *Mode* = 4.77, *SD* = 1.23, *range* 5.77, *skewness* = −0.42, *Kurtosis =* −0.34, and is presented in Figure 4.

**Table 2:** Study 1-means, standard deviations, and correlations with significance.

Variable	*M*	*SD*	1	2	3	4	5	6	7	8	9
1. Synchronicity awareness	2.26	0.99									
2. Synchronicity Meaning-Detecting	4.73	1.23	0.59**								
3. Extraversion	3.19	0.80	0.18**	0.20**							
4. Agreeableness	3.21	0.86	0.12*	0.17**	0.13**						
5. Conscientiousness	3.96	0.86	0.02	0.18**	0.07	0.12*					
6. Neuroticism	2.66	0.97	−0.06	−0.01	0.09	−0.35**	−0.25**				
7. Openness	3.42	0.94	0.39**	0.30**	0.06	0.04	0.06	0.03			
8. Presence of meaning	4.51	1.30	0.25**	0.42**	0.15**	0.26**	0.37**	−0.28**	0.20**		
9. Search for meaning	4.63	1.50	0.21**	0.42**	0.12*	0.03	0.08	0.02	0.16**	0.27**	
10. Tolerance for ambiguity	3.95	0.85	0.22**	0.18*	0.05	0.23**	0.22**	−0.41**	0.21**	0.25**	−0.02

*p < 0.05, **p < 0.01.

Next, we calculated Pearson correlations of each of the factors with the Big Five personality dimensions and tolerance for ambiguity and search for and presence of meaning in life. As can be seen in Table 2, and as predicted, synchronicity awareness and meaning-detecting scores were positively associated with extraversion (*r* = 0.18, *p* < 0.001; *r* = 0.20, *p* > 0.001, respectively), agreeableness (*r* = 0.12, *p* = 0.02; *r* = 0.17, *p* < 0.001, respectively), openness to experience (*r* = 0.39, *p* < 0.001; *r* = 0.30, *p* < 0.001, respectively), presence of meaning (*r* = 0.25, *p* < 0.001, *r* = 0.42, *p* < 0.001, respectively), search for meaning (*r* = 0.21, *p* < 0.001, *r* = 0.42, *p* < 0.001, respectively), and tolerance for ambiguity (*r* = 0.22, *p* < 0.001, *r* = 0.18, **p <* 0.*01, respectively). Synchronicity meaning-detecting scores were also positively associated with conscientiousness (*r* = 0.18, *p* < 0.001). The results support the contention that synchronicity awareness and meaning-detecting reflect related yet distinct constructs.

## Materials and methods: Study 2

Study 2 sought to expand the exploration of Study 1 by: (1) replicating its results in an independent and larger sample; (2) examining whether synchronicity scales are associated with measures of well-being; and (3) exploring the mediating roles of synchronicity awareness and meaning-detecting, optimism, and presence of meaning between search for meaning and life satisfaction.

### Participants

The second sample was another independent representative sample of the Israeli Jewish population. *A priori* power analysis of a linear multiple regression assessing a fixed model, R^2^ deviation from zero (G*Power; Faul et al., 2007), revealed a required total n = 92 to determine at least a medium effect (f 2 = 0.15, α = 0.05, 1 − β = 0.80 with five predictors). Therefore, data was obtained from 440 participants through the same online panel as in Study 1. Invitations were sent only to registered participants who did not take part in the first study. All participants on the panel had expressed their consent to participate in the study. Of the participants, 66.4% were women, 33.8% had a BA degree, and 24.9% had an MA degree or higher. Furthermore, 63.2% of the participants were married, 55.2% of them were secular, and the rest reported various levels of religiosity. Participants’ ages ranged from 18 to 85, with a mean age of 43.44 (*SD* = 15.59).

### Measures

#### Synchronicity awareness and meaning-detecting

The SAMD scale used in the second study was comprised of the two subscales that were developed in the first study: (a) synchronicity awareness (SA), and (b) synchronicity meaning-detecting (MD). Similar to Study 1, the SA subscale referred to awareness of the occurrence of synchronicity events. It involved 9 items using a 6-point scale (see Table 1) and included the same instructions as in Study 1. Cronbach’s α coefficient = 0.87, 95%CI [0.85, 0.89], McDonald’s ω coefficient = 0.90, 95%CI [0.84, 0.89]. The MD subscale referred to the meaning detected in the synchronicity experiences, involved 13 items using a 7-point scale (see Table 1), and included the same instructions as in Study 1. Cronbach’s α coefficient = 0.93, 95%CI [0.92, 0.94], McDonald’s ω coefficient = 0.94, 95%CI [0.92, 0.94].

#### Dispositional optimism

Optimism was measured by the validated Hebrew Optimism subscale of the LOT-R (Scheier and Carver, 1995; Wong and Lim, 2009; Palgi et al., 2011). The items are presented on a 5-point Likert-type scale, ranging from 0 (do not agree at all) to 4 (definitely agree). Sample items include: “In uncertain times, I usually expect the best,” “I am always optimistic about my future,” and “Overall, I expect more good things to happen to me than bad.” For the present sample, Cronbach’s α coefficient = 0.66, 95%CI [0.60, 0.70], McDonald’s ω coefficient = 0.62 95%CI [0.51, 0.68].

#### Meaning in life

This scale was used to assess the search for and presence of meaning in the individual’s life, with “search for” and “presence of” representing two subscales of the overall measure. Both subscales were rated using a 1 (absolutely untrue) to 7 (absolutely true) Likert scale (Steger et al., 2006). The present study used the Hebrew version of this questionnaire (Littman-Ovadia and Steger, 2010). The search for meaning subscale is comprised of five items (MLQ-S; e.g., “I am looking for something that makes my life feel meaningful” and “I am seeking a purpose or mission for my life”). Cronbach’s α coefficient = 0.88, 95%CI [0.86, 0.89], McDonald’s ω coefficient = 0.88 95%CI [0.85, 0.90]. The presence of meaning subscale is comprised of the remaining five items of the measure (MLQ-P; e.g., “I understand my life’s meaning” and “My life has no clear purpose”). Cronbach’s α coefficient = 0.85, 95%CI [0.83, 0.87], McDonald’s ω coefficient = 0.85, 95%CI [0.82, 0.88].

#### Depression

Depression was measured by the CES-D (Radloff, 1977). Participants reported depressive symptoms experienced during the past week (e.g., “I felt depressed”), measured on a 4-point scale from 0 (rarely or none of the time, less than 1 day) to 3 (all of the time, 5–7 days). We used the shortened (8 items) and validated to Hebrew version (Shmotkin and Keinan, 2011; Karim et al., 2015). Cronbach’s α coefficient = 0.89, 95%CI [0.88, 0.91], McDonald’s ω coefficient = 0.90, 95%CI [0.88, 0.91].

#### Anxiety

Anxiety was measured by the GAD-7, which consists of seven items measuring worry and anxiety symptoms (Spitzer et al., 2006). Each item is scored on a 4-point Likert scale (0–3) with total scores ranging from 0 to 21, with higher scores reflecting more severe anxiety. Scores above 10 are considered to be in the clinical range (Spitzer et al., 2006). We used the Hebrew validated version, which has shown good reliability and construct validity (e.g., Savitsky et al., 2020). Cronbach’s α coefficient = 0.87, 95%CI [0.85, 0.89], McDonald’s ω coefficient = 0.87, 95%CI [0.85, 0.89].

#### Satisfaction with life

To measure life satisfaction, we used the Hebrew-validated version of the 5-item SWLS, presented on a 7-point Likert-type scale, ranging from 1 (strongly disagree) to 7 (strongly agree), e.g., “The conditions of my life are excellent” (Diener et al., 1985; Anaby et al., 2010). For the present sample, Cronbach’s α coefficient = 0.87, 95%CI [0.85, 0.89], McDonald’s ω coefficient = 0.87 95%CI [0.85, 0.89].

### Data analysis

In order to validate the structure of the new SAMD scale, statistical analyses were conducted using R version 4.0.3 and R studio version 1.3.1093. To test the suggested statistical model, we employed confirmatory factor analysis and path analysis techniques using the lavaan package for R (Rosseel, 2012). Based on accepted practices (Hoyle and Panter, 1995; Mueller and Hancock, 2019), the fit of the model to the data was evaluated using several goodness of fit indices.

Three absolute fit indices were used: the χ^2^ statistic, the Root Mean Square Error of Approximation (RMSEA), and the Standardized Root Mean Squared Residual (SRMR). Four additional relative fit indices were used: the Normed Fit Index (NFI), the Comparative Fit Index (CFI), the Tucker-Lewis Index (TLI), and the Goodness of Fit Index (GFI). An expected cross validation index (ECVI) was calculated for the model. A non-significant χ^2^ statistic, RMSEA and SRMR scores below.06, as well as NFI, CFI, TLI, and GFI values above 0.95, indicate excellent fit, whereas SRMR values below 0.08 and NFI, CFI, TLI, and GFI above 0.90 indicate adequate fit. Lastly, ECVI were evaluated, since lower values are considered better.

Additionally, we compared the suggested model to a one-factor model to see if the two-factor model yielded a better fit to the data. After the final model was established, we tested the pairwise correlations between all the study variables and explored the research hypotheses using a path analysis model. As a part of the model test, we likewise tested for the significance of the indirect effects to determine if mediation effects would take place.

## Results: Study 1

### Preliminary analysis

A confirmatory factor analysis (CFA) was conducted on both subscales of the SAMD scale. The analysis demonstrated that the suggested two-factor model of the scale had an acceptable fit with the data, $\begin{array}{l} \chi^{2}\left(204\right)=571.35,p<0.001,NFI=0.89,TLI\\ \;=0.92,CFI=0.93,\mathrm{RMSEA}=0.06,GFI\\ \;=0.89,\mathrm{ECVI}=1.52,\mathrm{SRMR}=0.05\text{,} \end{array}$ and that all loadings exceeded 0.57 and were significant. In order to further validate the two-factor structure of the model, a one-factor model was also examined. The analysis demonstrated that the one-factor model had a poor fit with the data, $\begin{array}{l} \;\chi^{2}\left(209\right)=1382.06,p<0.001,NFI=0.74,\\ \;TLI=0.75,CFI=0.77,\mathrm{RMSEA}=0.11,\\ \;GFI=0.71,\mathrm{ECVI}=3.34,\mathrm{SRMR}=0.09 \end{array}$ . Furthermore, the two-factor model was found to be significantly better than a one-factor model fit ( $\Delta \chi^{2}\left(5\right)=810.71,p<0.001$ ).

These results substantiate the two-factor structure of the new scale. As can be seen in Table 3, all items indicate significant and positive estimates for two factors. Furthermore, the synchronicity awareness subscale had Cronbach’s $\begin{array}{l} \alpha =.87,95\%CI\;\left[0.85,0.89\right],\mathrm{McDonald},s\;\omega \;\\ \;\mathrm{coefficient}=0.90,95\%CI\;\left[0.84,0.89\right]\text{.} \end{array}$ The meaning-detection subscale had Cronbach’s $\begin{array}{l} \alpha =0.93,95\%CI\;\left[0.92,0.94\right],\mathrm{McDonald},s\;\omega \;\\ \;\mathrm{coefficient}=0.94,95\%CI\;\left[0.92,0.94\right]\text{.} \end{array}$ Thus the structure of the new scale was replicated and validated in a new sample.

**Table 3:** Study 2-synchronicity items factor loadings (CFA).

Scale	Scale item	b (SE)	Beta	Explained variance (%)
Synchronicity awareness	I felt that I was “in the right place, at the right time”	1.00	0.65	58%
	I ran into something or someone that I thought about in an unexpected place	0.96 (0.08)	0.63***	61%
	I ran into a situation or a personal encounter that opened up new opportunities	1.20 (0.10)	0.70***	51%
	I experienced an extraordinary synchronization of thought, behavior. Or words with another person	1.32 (0.11)	0.69***	52%
	I received an answer to a certain need I had in an unpredictable way (e.g., a partner, a job offer, or an apartment)	1.14 (0.11)	0.69***	54%
	I thought or dreamt about a person and then I met him\her somehow in the real world shortly afterwards	1.04 (0.09)	0.61***	63%
	I thought about a person and he\she contacted me unexpectedly shortly afterwards	1.06 (0.10)	0.62***	62%
	I thought about a particular idea and then I saw it as an external image (e.g., a quote, an ad, or a song)	1.38 (0.11)	0.71***	49%
	While in nature, I felt a strong sense of connection to the world	1.25 (0.12)	0.58***	67%
Synchronicity Meaning-Detecting	I believe that unexplained events enable new discovery and development	1.00	0.81	35%
	I find signs of inner feelings in the external stimuli in the world around me	0.96 (0.06)	0.73***	47%
	I find meaning in unexplained occurrences	0.70 (0.05)	0.61***	63%
	I believe that listening to internal and external occurrences enables new discoveries	0.88 (0.06)	0.69***	52%
	I sometimes feel that the environment “sends” me signals	1.08 (0.06)	0.78***	40%
	Following experiences I’ve had, I have a sense of deep knowing of myself and the world	1.02 (0.06)	0.78***	40%
	I am open to experiences that may not necessarily be explained by reason or causality	0.81 (0.06)	0.63***	60%
	I tend to be attentive to intuition in my everyday life	0.82 (0.05)	0.74***	46%
	I am curious about surprising events in my life	0.77 (0.05)	0.68***	53%
	I walk around in the world with a sense of awe and wonder from the opportunities and surprises that the world has to offer	0.90 (0.06)	0.68***	53%
	It happens that things related to issues I am concerned with suddenly appear more in my everyday life	0.89 (0.05)	0.76***	42%
	I believe that there is something to be learned from any event in life	0.65 (0.04)	0.65***	57%
	I tend to be attentive to physical and bodily sensations (e.g., goosebumps, pain, sense of warmth)	0.81 (0.06)	0.66***	57%

***p < 0.001.

As for the prevalence of synchronicity experiences, in the second sample (N = 440), 99% of the participants reported they experienced at least one or more such encounters (only 4 out of 440 reported they did not experience any of the given examples). As Table 4 shows, when asked to estimate the frequency of synchronicity experiences in their daily lives on a scale between “never” (0) to “all the time” (5), the average response of the sample ranged between “twice or more” (2) to “rarely” (3) (*M* = 2.29, *SD* = 0.99, *Mdn* = 2.22, *Mode* = 1.89, *range* 5.00, *skewness* = 0.031, *Kurtosis =* −0.37). The distribution of synchronicity awareness in the second sample is presented in Figure 3. As for the distribution of meaning-detection scores, we found that *M* = 4.74, *SD* = 1.21, *Mdn* = 4.81, *Mode* = 5.38, *range* 5.77, *skewness* = −0.038, *Kurtosis =* −0.38 (the distribution is presented in Figure 4). Taken together, the findings confirm the data from the first sample and suggest that synchronicity experiences are quite common and that the tendency to be aware of such experiences varies between individuals.

**Table 4:** Means, standard deviations, internal reliabilities and zero-order correlations of study 2.

Variable	*M*	*SD*	1	2	3	4	5	6	7
Synchronicity awareness	2.29	0.99							
Synchronicity meaning detecting	4.74	1.21	0.61**						
Optimism	3.53	0.61	0.18**	0.19**					
Search for meaning	4.53	1.40	0.26**	0.41**	−0.02				
Presence of meaning	4.81	1.23	0.20**	0.31**	0.40**	0.23**			
Depression	1.81	0.67	0.15**	0.15**	−0.37**	0.22**	−0.18**		
7. Anxiety	0.77	0.71	0.08	0.10*	−0.34**	0.14**	−0.17**	0.71**	
8. Life satisfaction	4.74	1.25	0.16**	0.12*	0.47**	0.01	0.48**	−0.42**	−0.35**

*p < 0.05, **p < 0.01.

After the revalidation of the new scales’ structure, our next step was to examine the suggested model hypotheses. First, to estimate the associations between the second study’s variables, we conducted an analysis of all pairwise Pearson correlations (see Table 4). The analysis revealed that life satisfaction was significantly and positively correlated with synchronicity awareness ( r=0.16,p<0.001 ), synchronicity meaning-detecting ( r=0.12,p=0.01 ), optimism ( r=0.47,p<0.001 ), and presence of meaning ( r=0.48,p<0.001 ). No correlation was found between search for meaning and life satisfaction ( r=0.01,p=0.78 ). Furthermore, life satisfaction was found to be negatively correlated both with depression ( r=−0.42,p<0.001 ) and with anxiety ( r=−0.35,p<0.001 ).

It was further found that synchronicity awareness is significantly and positively correlated with synchronicity meaning-detecting ( r=0.61,p<0.001 ), optimism ( r=0.18,p<0.001 ), search for meaning ( r=0.26,p<0.001 ), presence of meaning ( r=0.20,p<0.001 ), and depression ( r=0.15,p<0.001 ). Synchronicity meaning-detecting, in turn, was found to be significantly and positively correlated with optimism ( r=0.19,p<0.001 ), search for meaning ( r=0.41,p<0.001 ), presence of meaning ( r=0.31,p<0.001 ), and depression ( r=0.15,p=0.001 ). Optimism was further found to be correlated with presence of meaning ( r=0.40,p<0.001 ) but not with search for meaning ( r=−0.02,p=0.61 ). Optimism was, likewise, found to have negative correlations both with depression ( r=−0.37,p<0.001 ) and with anxiety ( r=−0.34,p<0.001 ).

Search for meaning was positively correlated with presence of meaning ( r=0.23,p<0.001 ), depression ( r=0.22,p<0.001 ), and anxiety ( r=0.14,p<0.001 ), while presence of meaning was found to have negative correlations both with depression ( r=−0.18,p<0.001 ) and with anxiety ( r=−0.17,p<0.001 ). Finally, depression and anxiety had a positive correlation ( r=.71,p<0.001 ).

Hence, as expected, search for meaning was positively associated with synchronicity awareness, which was positively associated with synchronicity meaning-detecting. Moreover, as predicted, the tendency to detect meaning in synchronicity experiences was positively correlated with both the presence of meaning in life and a sense of optimism, which were positively associated with life satisfaction.

### Test of indirect effects

**Figure 5:**
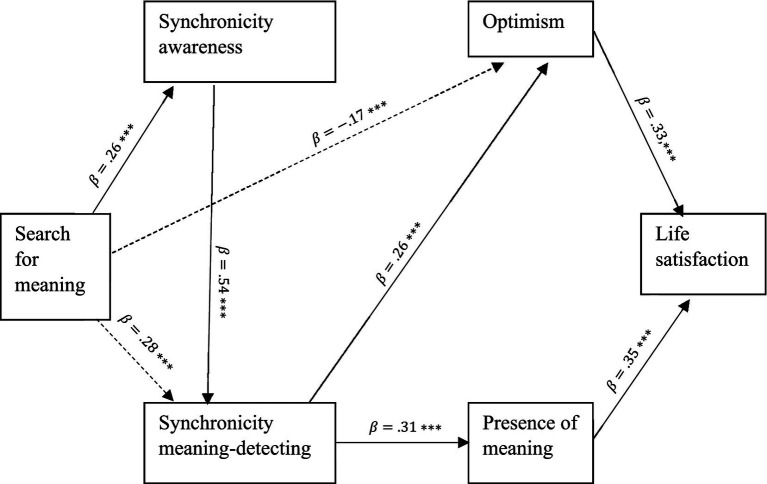
The model coefficients estimated *via* path analysis. Solid lines with arrows indicate paths in the original model. Dashed lines with arrows indicate paths added from modification indices. Numbers above lines indicate standardized path coefficients and significance values. ***p < 0.001.

We used path analysis (see Figure 1 for the theoretical model). Following Hayes, 2017) multiple mediation analysis outline, our hypotheses were tested using 10,000 bootstrapped samples and 95% confidence intervals. The initial model’s fit (marked by solid lines with arrows in Figure 5) did not reach an acceptable level. Thus, modification indices were inspected for theoretically viable paths (dashed lines with arrows in Figure 5 indicate paths added to the initial model). The final model had fit indices that met criteria for good to excellent model fit: $\begin{array}{l} \chi^{2}\left(6\right)=17.12,p=0.01,\mathrm{RMSEA}=0.06,\\ \;NFI=0.97,TLI=0.95,CFI=.98,\\ \mathrm{SRMR}=0.03,GFI=0.99,\mathrm{ECVI}=0.10 \end{array}$ . All direct paths in the model were significant and the explained variance in the model for synchronicity awareness was $R^{2}=0.066$ , for synchronicity meaning-detecting was $R^{2}=0.44$ , for optimism was $R^{2}=0.061$ , for presence of meaning was $R^{2}=0.10\text{,}$ and for life satisfaction was $R^{2}=0.327$ .

Table 5 details all standardized, unstandardized, and significance of the model coefficients. Furthermore, testing for the indirect effects between search for meaning and life satisfaction in the model *via* the bootstrapping procedure (Hayes, 2009) yielded five significant indirect effects, indicating significant mediation effects. Specifically, the sequential indirect effect of search for meaning on life satisfaction through synchronicity awareness, synchronicity meaning-detecting, and optimism was found to be significant: $b=0.01,se=0.003,95\%CI\left[0.006,0.019\right]$ .

**Table 5:** Standardized and unstandardized coefficients in the path analysis model.

Variable	b (SE)	β	*p*	*95%CI*
DV: Synchronicity awareness $R^{2}=0.07$				
Search for meaning	0.18 (0.03)	0.26	**<0.001**	[0.12, 0.24]
DV: Synchronicity meaning-detecting $R^{2}=0.44$				
Search for meaning	0.24 (0.03)	0.28	**<0.001**	[0.17, 0.31]
Synchronicity awareness	0.66 (0.04)	0.54	**<0.001**	[0.57, 0.31]
DV: Optimism $R^{2}=0.06$				
Search for meaning	−0.08 (0.02)	−0.17	**0.001**	[−0.12, −0.03]
Synchronicity meaning-detecting	0.13 (0.03)	0.26	**<0.001**	[0.08, 0.19]
DV: Presence of meaning $R^{2}=0.10$				
Synchronicity meaning-detecting	0.32 (0.05)	0.31	**<0.001**	[0.24, 0.42]
DV: Life satisfaction $R^{2}=0.33$				
Optimism	0.68 (0.09)	0.33	**<0.001**	[0.50, 0.85]
Presence of meaning	0.35(0.05)	0.35	**<0.001**	[0.26, 0.45]

The sequential indirect effect of search for meaning on life satisfaction through synchronicity meaning-detecting and optimism was found to be significant: $b=0.021,se=0.006,95\%CI\left[0.012,0,036\right]$ . The sequential indirect effect of search for meaning on life satisfaction through synchronicity meaning-detecting and presence of meaning was found to be significant: $b=0.027,se=0.007,95\%CI\left[0.015,0.043\right]$ . The sequential indirect effect of search for meaning on life satisfaction through synchronicity awareness, synchronicity meaning-detecting, and presence of meaning was found to be significant: $b=0.013,se=0.004,95\%CI\left[0.008,0.022\right]$ . Also, the indirect effect of search for meaning on life satisfaction through optimism was found to be significant: $b=-0.051,se=0.018,95\%CI\left[-0.09,-0.02\right]$ . See Table 6 and Figure 5 for the final model paths with their standardized coefficients.

**Table 6:** Indirect effects from search for meaning on life satisfaction testing both parallel and sequential mediation effects.

Mediator 1	Mediator 2	Mediator 3	Boot ab	95% CI
Optimism			−0.051 (0.018)	[−0.09, −0.02]
Synchronicity awareness	Synchronicity meaning-detecting	Optimism	0.02 (0.005)	[0.008, 0.026]
Synchronicity meaning-detecting	Optimism		0.021 (0.006)	[0.012, 0.036]
Synchronicity meaning-detecting	Presence of meaning		0.027 (0.007)	[0.015, 0.043]
Synchronicity awareness	Synchronicity meaning-detecting	Presence of meaning	0.013 (0.004)	[0.008, 0.022]

Boot *ab* refers to bootstrapped indirect effect; bootstrap sample size *n* = 10,000. Unstandardized regression coefficients reported are based on bias-corrected and accelerated 95% confidence intervals (CIs). CIs that do not include zero indicate significant effects.

In sum, Study 2 further validated the internal structure of the SAMD scale in an independent and larger sample. In addition, as suggested in the model, search for meaning was positively associated with synchronicity awareness (*a*), which was associated with higher synchronicity meaning-detecting (*b*). In addition, higher tendency to detect meaning in synchronicity experiences was positively associated with both presence of meaning in life (*c*) and a sense of optimism (*d*), and each in turn was related to enhanced life satisfaction (*e* and *f*).

## Discussion

The first aim of the current study (encompassing the two individual studies described here) was to develop a valid tool to assess individual differences in the tendency to be aware of synchronicity experiences and to detect meaning in them. In the two samples, a vast majority of the participants reported experiencing at least one synchronicity event. This finding extends previous research documenting the scope and prevalence of the phenomenon among nonclinical, general population samples (e.g., Bright et al., 2005; Fach et al., 2013), suggesting that awareness of synchronicity experiences is rather widespread.

More significantly, the present study involved the development and validation of a measure for assessing individual difference in the awareness and meaning-detecting of synchronicity experiences: the SAMD scale. The two-factor structure of the questionnaire that emerged from the data supports the “bottom-up” model of REM (Russo-Netzer and Icekson, 2020). The two factors demonstrated high internal reliability and appear to capture distinct yet related aspects of the synchronicity experience phenomenon.

Another contribution of the present study is a better understanding of the construct of synchronicity awareness by adding the dimension of meaning-detecting. While previous studies focused mainly on the frequency of noticing such events (e.g., Coleman et al., 2009; Fach et al., 2013), the present study also explored the meaning individuals attribute to such experiences. Furthermore, the findings suggest that synchronicity awareness and meaning-detecting are positively associated with openness to experience and tolerance for ambiguity. This finding may correspond with observations from recent brain studies, suggesting that the tendency to experience meaningful coincidence was negatively correlated with gray matter in the brain in regions involved in causality detection and emotional control (Unger et al., 2021). This delineates an interesting direction for further research exploring the neural characteristics and personality traits of individuals who are more prone to be aware of and make sense of synchronicity experiences.

The second aim of the present study was to extend previous knowledge on the complex relation between the search for meaning and life satisfaction, pointing out the role of synchronicity awareness and meaning-detecting as possible mediators. Previous studies revealed a complex relationship between the search for meaning in life and well-being (e.g., Steger et al., 2011; Yek et al., 2017). The results of the present model suggest that individuals who search for meaning and are open to synchronicity events and manage to make sense of them may experience more meaning and optimism, which eventually may contribute to greater life satisfaction. These findings correspond with previous studies suggesting that the search for meaning in life may contribute to life satisfaction (e.g., Datu, 2015; Russo-Netzer, 2019) under certain circumstances. More specifically, this study adds an underexplored, potential link in the chain between these two constructs, suggesting that the interplay between these constructs is not straightforward, thus calling future studies to further explore the contribution of synchronicity as well as other potential mediators.

The findings also indicate that, as was found in an ample number of previous studies (Daukantaitė and Zukauskiene, 2012; Zhang et al., 2014; Busseri and Choma, 2016), higher optimism is positively correlated with greater life satisfaction. More importantly, we also found that optimism mediates between meaning-detecting of synchronicity experiences and life satisfaction. It is possible that when individuals manage to detect meaning in unexpected, unexplained experiences in their environment, they regain a sense of order and coherence that may cultivate their optimism. Along these lines, it was found that when contemplating future decisions in business and leadership, individuals have utilized synchronicities (Laveman, 2014; Beitman, 2016; Cristofaro, 2021). This direction extends previous limited knowledge on the ways in which people can enhance positive expectations of the future (Malouff, 2017) and may open up new directions for further research and practice.

### Limitations and suggestions for future research

The present study has several limitations that should be taken into consideration. First, the study data was collected from a single source: self-report surveys, a method that, although considered suitable for assessing subjective experiences (e.g., Sheldon and Lyubomirsky, 2007), could also lead to some biases in participants’ responses. To cope with this limitation, we used procedural design methods [confidentiality and anonymity, separate questionnaire sections and instructions, etc.; Podsakoff et al., 2003)]. Future research could involve other sources (such as brain and behavioral measures) in order to provide further evidence beyond self-reporting methods.

Second, the present findings are correlational and are based on cross-sectional research, thus causal directionality implied should be examined with longitudinal designs or intervention and experimental research to further validate and refine the newly developed measure of synchronicity awareness and meaning-detecting (the SAMD scale) and its implications. For example, future research could use daily diary methods (e.g., Rodríguez-Carvajal et al., 2019).

Lastly, the scope of the present study mainly focused on the potential positive aspects of synchronicity awareness and meaning-detecting. Yet, it should be noted that as we still know rather little about this complex construct and the boundary conditions that may hinder its beneficial outcomes, thus it may be worthwhile to explore when such processes reflect healthy and non-healthy experiences. Given the positive, yet weak, association that was found in the present study between the two factors of synchronicity scale and depression, it may be possible that overinterpretation or excessive rumination over unexpected events may trigger a distorted sense of meaning and may lead to undesirable experiences. For example, it was previously suggested that unregulated explanatory models regarding coincidence experiences may lead to psychopathology processes such as paranoia or magical thinking (e.g., Beitman et al., 2010).

Overall, despite these limitations, this study extends existing literature of clinical reports and case studies on the phenomenon of synchronicity by taking a step further to provide possible directions to better understand the underlying mechanisms leading from one’s search for meaning to life satisfaction. The results imply potential applied pathways for the development of therapeutic, organizational, and educational practical interventions to enhance well-being. Such a mindset may support individuals in coping with the challenges of our changing world, where uncertainty and complexity appear to be a significant part of our day-to-day reality.

## Data availability statement

The raw data supporting the conclusions of this article will be made available by the authors, without undue reservation.

## Ethics statement

The studies involving human participants were reviewed and approved by University of Haifa. All procedures performed in studies involving human participants were in accordance with the ethical standards of the institutional research committee and with the 1964 Helsinki declaration and its later amendments or comparable ethical standards. The participants provided their written informed consent to participate in this study.

## Author contributions

PR-N and TI have made substantial contributions to the conception of the study, the acquisition, analysis, and interpretation of the research data, and in preparing the manuscript for publication. All authors contributed to the article and approved the submitted version.

## Conflict of interest

The authors declare that the research was conducted in the absence of any commercial or financial relationships that could be construed as a potential conflict of interest.

## Publisher’s note

All claims expressed in this article are solely those of the authors and do not necessarily represent those of their affiliated organizations, or those of the publisher, the editors and the reviewers. Any product that may be evaluated in this article, or claim that may be made by its manufacturer, is not guaranteed or endorsed by the publisher.
