# Supplementary material for: An underexplored pathway to life satisfaction: The development and validation of the synchronicity awareness and meaning-detecting scale
Source: Front Psychol. 2023 Jan 16;13:1053296. doi: 10.3389/fpsyg.2022.1053296 (PMC9885050; doi:10.3389/fpsyg.2022.1053296)
Supplement: Supplementary file 1 [file Table_1.docx]

**Supplementary materials**

**An Underexplored Pathway to Life Satisfaction: The Development and Validation of the Synchronicity Awareness and Meaning-Detecting (SAMD) Scale**

Pninit Russo-Netzer & Tamar Icekson

**Table 1**

Demographic Characteristics of Participants in Study 1

| Full sample (*N =* 410) | | Sample 2 (*N =* 212) | | Sample 1 (*N =* 198) | |  |
| --- | --- | --- | --- | --- | --- | --- |
| *%* | *n* | *%* | *n* | % | *n* |  |
|  |  |  |  |  |  | Gender |
| 59.51% | 244 | 50% | 106 | 69.7% | 138 | Female |
| 40.49% | 166 | 50% | 106 | 30.3% | 60 | Male |
|  |  |  |  |  |  | Marital Status |
| 35.4% | 145 | 24.5% | 52 | 45% | 93 | Single |
| 55.6% | 228 | 62.7% | 133 | 47% | 95 | Married/partnered |
| 9% | 37 | 12.7% | 27 | 5.1% | 10 | Divorced/Widowed |
|  |  |  |  |  |  | Highest educational level |
| 39.3% | 161 | 53.8% | 114 | 23.7% | 47 | High school/Yeshiva |
| 35.4% | 145 | 32.5% | 69 | 38.4% | 76 | Bachelor's degree |
| 18.8% | 77 | 13.7% | 29 | 24.25% | 48 | Master's degree or higher |
| 6.6% | 6 | 0% | 0 | 13.6% | 6 | Other |
|  |  |  |  |  |  | Religiosity (Jewish) |
| 53.9% | 221 | 45.8% | 97 | 62.6% | 124 | Secular |
| 29.5% | 121 | 32.1% | 68 | 26.8% | 53 | Traditional |
| 12% | 49 | 13.2% | 28 | 10.6% | 21 | Orthodox |
| 4.6% | 19 | 9% | 19 | 0% | 0 | Ultra - Orthodox |
| *SD* | *Mean* | *SD* | *Mean* | *SD* | *Mean* |  |
| 15.67 | 39.22 | 7.48 | 29.70 | 1.04 | 34 | Age |
